# Prevalence and Associated Factors of Attention-Deficit Hyperactivity Disorder among Children Aged 6–17 Years in North Eastern Ethiopia

**DOI:** 10.4314/ejhs.v32i2.13

**Published:** 2022-03

**Authors:** Getaneh Baye Mulu, Ahmed Yimam Mohammed, Worku Misganaw Kebede, Bantalem Tilaye Atinafu, Fetene Nigussie Tarekegn, Hana Nigussie Teshome, Mesfin Tadese, Abate Dargie Wubetu

**Affiliations:** 1 Department of Nursing, College of Health Science, Debre Berhan University, Debre Berhan, Ethiopia; 2 Department of Psychiatry, College of Health Science, Debre Berhan University, Debre Berhan, Ethiopia; 3 Department of Midwifery, College of Health Science, Debre Berhan University, Debre Berhan, Ethiopia; 4 Department of Anatomy, College of Medicine, Debre Berhan University, Debre Berhan, Ethiopia; 5 The National Tobacco Enterprise (Ethiopia) Share Company (NTE), Northeastern, Ethiopia

**Keywords:** Associated factors, Children, Attention-deficit hyperactivity disorder, Ethiopia

## Abstract

**Background:**

Attention-deficit hyperactivity disorder is one of the public neurodevelopmental disorders characterized by impulsivity and restlessness or hyperactivity. This study aimed to assess the prevalence of Attention-deficit hyperactivity disorder and its associated factors among children aged 6 to 17 years in Shewa Robit town, Northeastern Ethiopia, 2020.

**Methods:**

A community-based cross-sectional study was conducted among 365 children aged 6–17 years from Feb 1-March 30, 2020, at Shewa Robit town. Systematic random sampling was employed to select study participants. Data were collected by interview using structured and pretested questionnaires. Finally, data was entered using Epi-data 4.2 and analyzed using SPSS version 25. Bivariable and multivariable binary logistic regression analysis was conducted to identify associated factors of attention deficit hyperactivity disorder. Odds ratios with 95% CI were calculated, and variables having a p-value < 0.05 were considered statistically significant.

**Result:**

The prevalence of ADHD among children aged 6 to 17 years was 13%. Financial crises [AOR 4.76(95% CI 1.51–15.05)], children a previous history of the mental problem [AOR 8.45(95% CI 1.24–57.43)], C/S delivery [AOR 6.38(95% CI 1.26–32.26)] and substance use in life [AOR 2.43(95% CI 1.09–5.43)] were significantly associated with attention deficit hyperactivity disorder.

**Conclusion:**

The prevalence of ADHD in children 6 to 17 years old was high (13%). Financial crises, children's history of mental disorders, C/S delivery, and lifetime substance use were significantly associated with attention deficit hyperactivity disorder. Therefore, particular attention should be given to mothers and children with significant factors.

## Introduction

Attention-deficit hyperactivity disorder (ADHD) is a commonest neurodevelopmental disorder characterized by impulsivity and restlessness, affecting all age groups with variation in prevalence rate and symptom presentation in children, adolescents, and adults. According to DSM 1V criteria, ADHD is defined as inattention, including increased distractibility and difficulty sustaining attention, poor impulse control and decreased self-inhibitory capacity, and motor overactivity and restlessness in a child than expected for someone of that age and developmental level. Global epidemiological prevalence of 5.3% is one of the most common mental illnesses in childhood and adolescence (1). Furthermore, research shows that the prevalence of ADHD ranges between 0.9% and 20%, which showed the consistency of estimates and the validity of diagnosis due to assessment tools and diagnostic criteria (2). However, the overall prevalence of Children and adolescents between 1994 and 2010 ranged between 5.9% and 7.1%. In America, approximately 11% of children ages 4–17 years were diagnosed with ADHD in 2011(3). In addition, the pooled prevalence of ADHD in China was 6.26 % which is consistent with the global prevalence (4).

Research conducted in psychiatry advisory indicated that from 1997/1998 to 2015/2016, there was an increase in the prevalence of ADHD among children and adolescents. The research found that 7.9% of children and adolescents were reported to have been diagnosed with ADHD. In 2015/2016, the reported prevalence of diagnosed ADHD was 10.2% (5). The study result of an observational cross-sectional study in Saudi Arabia showed that the prevalence of ADHD among 1000 primary school children was 3.4 % (6). The prevalence of ADHD in Africa varied from 6% studied between 7–9 age school children in Congo and 11.7% in Uganda, respectively (7, 8). Despite ADHD having severe health and economic impact on the communities and nations as a wide, in Ethiopia, few studies were conducted, and the mechanism also not well understood. There is a vast difference in the prevalence of ADHD among studies conducted in different parts of Ethiopia, for example, in the Guji zone (6%), Mekelle town (18.5%), and in the Oromia region (13.7%) (9–11). Most of the time, ADHD is revealed before seven years old and occurs with one or more comorbid psychiatric problems like epilepsy and autism spectrum disorder (12, 13). ADHD results from complex interactions between genetic, environmental, and neurobiological factors (14). Being preterm, unable to breastfeed, history of child mental problems, medical problems during pregnancy, and low family socioeconomic status were significant factors associated with ADHD (10, 11). A study conducted in the Tigray region showed that family history of psychiatric illness and family history of medical illness was associated with ADHD (15) Although childhood ADHD research was extensively conducted in Europe and North America, very few studies are available in the sub-Saharan region, particularly Ethiopia. Therefore, this study aimed to investigate the prevalence of ADHD and its associated factors among children aged 6 to 17 years in Shewa Robit town Northeast Ethiopia. Furthermore, this study was used for evidence-based practice on maternal care services preventive and curative care of ADHD.

## Methods and Materials

**Study area and period**: This study was conducted in Shewa Robit town, Northeastern Ethiopia, from February 1 to March 30, 2020. Shewa Robit town is one of the 24 Woredas in the North Shoa Zone of the Amhara region. The town is located 225 km north of the capital city of Ethiopia, Addis Ababa. According to the woreda health sector annual plan of 2019, Shewa Robit has a total population of 54,306, of whom 26,176 are a man and 28,130 women (16). In Shewa Robit Town, nine kebeles, one health center, two hospitals, four private clinics, and six pharmacies are found.

**Study design**: A community-based cross-sectional study was employed among children 6 to 17 years old to determine the prevalence and associated factors of ADHD.

**Source population**: Children aged 6 to 17 years who live in Shewa Robit town

**Study population**: The study population was randomly selected children from the source population who fulfilled the inclusion criteria. Children between 6 and 17 years are found in each household, and actual data collection is taken. Children with severe disease and neurological handicap were excluded.

**Sample size determination**: The sample size was determined using a single population proportion formula by considering a study done in Mekele, Ethiopia, 18.5% (10); 95% confidence level, 5% of desired precision, 1.5 design effect, and 5% nonresponse rates were considered. Thus, the final sample size was 365.

**Sampling technique and procedure**: The psychiatry nurses approached the guardians/parents for consent to participate in the study. Among study participants aged 12 or older without severe intellectual disability, assent was considered to participate in the study. The three psychiatry nurses interviewed the parents or the study participants (those who gave assent) using a pretested questionnaire written in English, administered in the Amharic language. In addition, parents were asked to recall symptoms from a list of criteria for diagnosing ADHD exhibited by their children at home.

A multi-stage sampling technique was used for the selection of sampling units. First, it was assumed that the population of Shewa Robit town is homogenous. Therefore, the town has nine kebele with 8500 households. From this, three kebeles were selected by a simple random sampling technique. Those three kebeles in Shewa Robit town have 3000 households. Of those, 2200 households have children aged 6 to 17 years. Three hundred sixty-five households with at least one child aged 6 to 17 years were allocated for three selected kebeles using systematic random sampling techniques every six intervals (2200/365= 6) by proportional allocation. The household sampling frame was obtained from Health extension workers found in each kebele of Shewa Robit town. Only one child was selected using the lottery method during the availability of more than one child in a household. ADHD was screened using the disruptive behavior disorder rating scale (11).

Attention deficit hyperactivity disorder was the dependent varibales.

The following are taken as independent variables.

**socio-demographic factors** (child age, sex, maternal age at pregnancy time, a religion of child, ethnicity, currently living with, family educational status, family income level, number of household members)

**Psychosocial factors**: stressful life events

Substance-related factors (substance use during pregnancy time, lifetime substance use)

**Clinical factors** (current history of psychiatric disorder in the child, history of mental illness, family history of mental illness, history of chronic medical/physical illness of the child

**Obstetric and neonatal factors** (any complication during pregnancy, complicated delivery, low birth weight/prematurity, intrauterine growth retardation, low Apgar score

**Data collection tools**: The data were collected by interviews with the parents/caregivers using structured and pretested questionnaires. The interview was conducted by three trained psychiatry nurses' working in Shewa Robit public hospital and two supervisors. The questionnaire had five parts socio-demographic characteristics, psychosocial factors, substance-related factors, clinical factors, and obstetric and neonatal factors. In addition, the disruptive behavior disorders rating scale was completed for each study participant to identify the children who were likely to have ADHD symptoms (11). Finally, the psychiatry professional confirmed the diagnosis of ADHD using the Mini International Neuropsychiatric Interview for Children and Adolescents (MINI Kid) version 6.0, a tool based on DSM IV criteria for diagnosis of psychiatric conditions (17).

**The following operational definitions were used for this study.**

**Stress entire life event**: the presence of stressful life events explained by experiencing one or more stressful life events in the last six months.

**Use of substance during pregnancy**: use at least one specified substance (alcohol, chat, cigarette) throughout the child's gestation.

**Lifetime substance use**: this study defined **the use of any substance** (cigarette, Khat, alcohol) at least once throughout their lifetime

**Low APGAR score**: Our study defined it as when the child fails to cry soon after delivery or fails to suck breast milk 30 minutes after delivery.

**Data quality control measures**: Experts in related fields evaluated the questionnaire. The questionnaire was first prepared in English and translated to Amharic and back to English to check for language consistency. Then, the data collection instrument was pretested on 5% of the sample size in Shewa Robit town other than selected kebeles to avoid information contamination. The pretest was used to check for language clarity, appropriateness of data collection tools, estimate time required, and the necessary amendments were considered. In addition, training was given concerning the data collection tool and process for data collectors. Finally, three psychiatry nurses collected the data. During the data collection time, close supervision was carried out by supervisors and investigators to ensure the quality of the data. Finally, the supervisor and investigator checked all the collected data for completeness and consistency. Consistency was examined through a random selection of questionnaires.

**Data processing and analysis**: Before analysis, data were cleaned, edited, and coded. Data was entered using Epi-Data version 4.2 and analyzed using SPSS 25 statistical software. Based on the nature of variables frequency distribution, summary statistics are used to explain variables' characteristics. Independent variables having a P-value less than or equal to 0.25 in the bivariable analysis were exported to the multivariable logistic regression model. In multivariable regression P-value, less than 0.05 was considered statistically significant. Finally, data were presented in text, table, and graphs.

**Ethics approval**: Helsinki's declaration for medical research involving human subjects was followed. Ethical clearance was obtained from the Institutional Health Research Review Committee (Ref. No/NURS/19/2021) of the college of health and medicine, Debre Berhan University. Ethical clearance was obtained from the Institutional Health Research Review Committee of Debre Berhan University, college of health science. A permission letter was written for each study health institution, and a permission letter was taken from the study institution administrator. Written informed consent was taken from each study participant.

## Results

**Socio-demographic characteristics**: A total of 355 study participants were included in this study, which made the response rate 97.3%. More than half of the participants, 205(57.7%), were between ages 6–11 years. The mean ages of participants were (10.99 ± 3.036). Nearly half of the respondents (51.5%) were male. The majority (83.7%) of the participants belonged to the Amhara in ethnicity, and 43.4% were orthodox religious followers. Of the respondents, 60% live within a family size of 2–4 members. Children whose parents earn more than 2000 birr per month were 57.5%, and whose mother and father had a diploma and above by educational background were 14.6%, and 27.9%, respectively (Table 1).

**Table 1:** Socio-demographic characteristics of study participants at Shewa Robit town Northeastern Ethiopia, 2020

Variables	Frequency (n= 355)	Percent
Age in year		
6–11	205	57.7
12–17	150	42.3
Sex		
Male	183	51.5
Female	172	48.5
Ethnicity		
Oromo	5	1.4
Amhara	297	83.7
Tigray	43	12.1
Argoba	5	1.4
Others	5	1.4
Religion		
Orthodox	154	43.4
Muslim	138	38.9
Protestant	63	17.7
Other		
Family size		
2–4	213	60
5–6	115	32.4
≥ 7	27	7.6
Household income		
<500	16	4.5
500–1000	89	25.1
1001–2000	46	13
>2000	204	57.5
Fathers' education		
Unable to read and write	52	14.6
Able to read and write	69	19.4
Primary school	52	14.6
Secondary school	83	23.4
Diploma	46	13
Degree and above	53	14.9
Mothers' education		
Unable to read and write	86	24.2
Able to read and write	27	7.6
Primary school	101	28.5
Secondary school	89	25.1
Diploma	42	11.8
Degree and above	10	2.8
Parental marital status		
Married	238	67
Divorced	36	10.1
Single/separated/widowed	81	22.8

**Prevalence of Attention-Deficit Hyperactivity Disorder**: The total prevalence of ADHD among children aged 6 to 17 years was 13%. The prevalence of ADHD in male and female children was 15.84% and 9.8%, respectively. The highest prevalence of ADHD was among 7and 9 years old children each (18%). The younger the birth order, the higher the prevalence of ADHD. The proportion of ADHD subtype in this study was inattentive type 10.8% with slightly higher in male 1.5: 1, the hyperactivity-impulsivity type represented 14.15% with a male-female ratio of 2.25:1, and the combined type represented 25% with a malefemale ratio of 1.55:1

**Figure 1:**
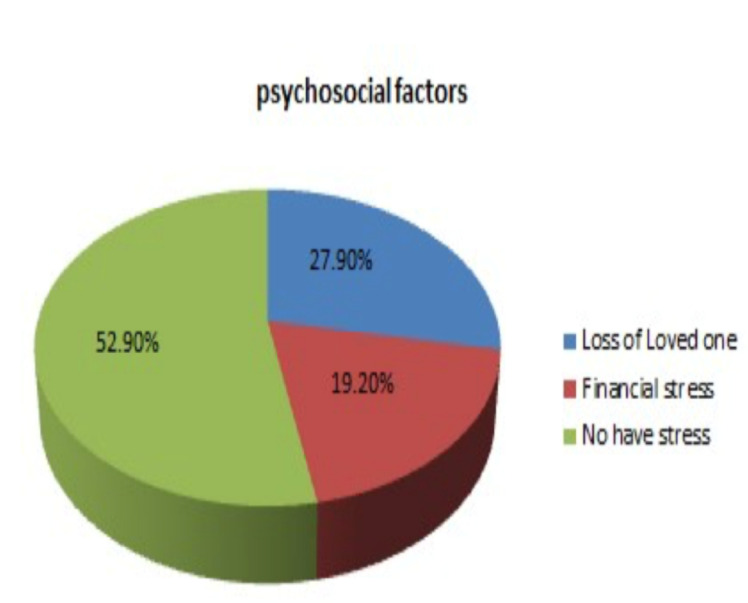
Distribution of study participants by Psychosocial factors at Shewa Robit town Northeastern Ethiopia, 2020.

**Psychosocial factors**: More than half of the study participants (52.9%) didn't experience psychosocial stress. Related to stress full life events, 27.9% of study participants reported losing a loved one. Of this 12.5% due to serious illness, 5.6% reported a loss of friends, and 10.1% reported a loss of family members. Sixty-eight (19.2%) have gone through financial stress within the last six months (Figure 1).

**Substance use-related factors**: Among 355 mothers of study subjects, 19.4% used substances; from these substances, 1.4% smoked, 93% chewed Khat, and 8.7% drunk alcoholic beverages at least once in life. Lifetime substance use during pregnancy was also reported among 84.5% of mothers. In addition, 0.8% smoke cigarettes, 8.7% are drunk, and 5.9% chewed Khat.

**Clinical factors in children and family**: Of the total 355 children aged 6 to 17 years who participated in the study, 1.4% had been diagnosed with a comorbid psychiatric disorder. In addition, family history of mental health problems and medical or physical disorders reported 5.1% and 2.5%, respectively.

**Neonatal and obstetric problems**: The study participants of mothers who have faced pregnancy complications were 8.2% from this vaginal bleeding, 1.4%, preeclampsia (3.4%), sexually transmitted disease 1.7%, and others (1.7%). The majority of mothers from the study participants gave birth through SVD, 284 (80%) mode of delivery, whereas 71(20%) of mothers gave birth by others (instrumental and C/S) (Table 2).

**Table 2:** Frequency distribution of participants by neonatal and obstetric factors at Shewa Robit town, Northeastern Ethiopia, 2020

Variable	Frequency (n= 355)	Percent
Maternal factor		
Vaginal Bleeding	5	1.4
Preeclampsia	12	3.4
sexually transmitted disease	6	1.7
Others	6	1.7
Mode of delivery		
SVD	284	80
Instrumental	40	11.3
C/S	31	8.7
Neonatal factor		
Preterm		
Yes	7	2
No	348	98
Perceived LBW		
Yes	71	20
No	284	80
Did not cry soon after birth		
Yes	48	13.5
No	307	86.5
Problem with breastfeeding		
Yes	37	10.4
No	318	89.6
A problem soon after birth		
Yes	63	17.7
No	292	82.3

**Factors Associated with ADHD**: Binary logistic regression analysis examined the association between ADHD and each determinant factor. After adjusting all factors having p-value ≤ 0.25 on bivariate analysis, multivariable logistic regression analysis was done, and the following variable were found significantly associated (P <0.05).

Children who reported a history of previously diagnosed psychiatric illness had almost nine times higher odds of experiencing ADHD than those who did not report [AOR 8.45(95% CI 1.24–57.43)]. Among the neonatal and obstetric problems, children who delivered using cesarean section had higher odds of experiencing ADHD than those who delivered spontaneously [AOR 6.38 (95% CI 1.26–32.26)]. Maternal substance use during pregnancy was also associated with increasing the odds of having ADHD compared to those who had no substance use history [AOR 2.43(95% CI 1.09–5.43)] (Table 3).

**Table 3:** Bivariable and multivariable logistic regression analysis of factors associated with ADHD among children age 6 to 17 years in Shewa Robit town, Northeastern, Ethiopia

Variable	ADHD		COR (95%CI)	AOR (95%CI)
	Yes=46	No =309		
	N (%)	N (%)		
**Fathers' education**				
Unable to read and write	2(4.3)	50(16.2)	0.26(0.05–1.33)	5.27(0.91–30.55)
Able to read and write	8(17.4)	61(19.7)	0.86(0.29–2.55)	1.38(0.43–4.48
Primary school	10(21.7)	42(13.6)	1.57(0.55–4.48)	1.04(0.33–3.24)
Secondary school	16(34.8)	67(21.7)	1.57(0.60–4.12)	0.71(0.26–1.98)
Diploma	3(6.5)	43(13.9)	0.46(0.11–1.89)	2.95(0.66–13.21)
Degree and above	7(15.2)	46(14.9)	1	1
**Child history of mental illness**				
Yes	3(6.5)	2(0.6)	0.09(0.15–0.58)	8.45(1.24–57.4) *
No	43(93.5)	307(99.4)	1	1
**Neonatal problems**				
Yes	11(23.9)	52(16.8)	0.64(0.31–1.35)	1.48(0.65–3.37)
No	35(72.1)	257(83.2)	1	1
Preterm				
Yes	2(4.3)	5(1.6)	0.36(0.68–1.92)	1.94(0.32–11.97)
No	44(95.7)	304(98.4)	1	1
**Mode of delivery**				
SVD	35(76.1)	249(80.6)	1	1
Instrumental	3(6.5)	37(12)	0.58(0.17–1.97)	2.34(0.89–6.21)
C/S	8(17.4)	23(7.4)	2.475(1.03–5.96)	6.38(1.26–32.3) *
**Lifetime Substance use**				
Yes	14(30.4)	55(17.8)	2.02(1.01–4.04)	2.43(1.09–5.43) *
No	32(69.6)	254(82.2)	1	1

* Statically significant at P value ≤ 0.05, SVD; spontaneous vaginal delivery, C/S: cesarian section

## Discussion

ADHD remains a significant cause of cognitive and social impairment among children. Hence, this study was conducted on prevalence and associated factors of Attention-deficit hyperactivity disorder among children aged 6 to 17 years at Shewa Robit town. Factors significantly associated with ADHD were; financial crises, children's previous history of mental problems, C/S delivery, and substance use.

According to this study, the total prevalence of ADHD was 13% among children aged 6 to 17 years old. This result is nearly similar to the findings done in Ethiopia Jimma, which was 13.7%, Uganda 11.7%, and Jidda 11.6 % (8, 19, 20).

On the contrary, the current study finding was higher than the studies done in Girja district rural Ethiopia, which was 7.3% (9) in Nairobi 6.3% (21), China 9.11% (22), and Trabzon 8.6% (23). This might be because the current research excludes the impairment criteria of attention deficit hyperactivity disorder. The US reported that the prevalence of ADHD without impairment criteria was 16.1% and 6.8% when impairment criteria were considered (5).

On the other hand, this finding was lower than the study conducted in Mekelle Tigray Ethiopia 18.5% (10) and Egypt showed in teacher rating and parent rating 21.8% and 16.2%, respectively (24). The possible reason for the discrepancy might be that the instruments used in the current study justify the high prevalence rates of ADHD than studies conducted based on DSM-III or ICD criteria. In addition, the study setting is another reason for the discrepancy in the finding. Therefore, the prevalence of ADHD among children in primary school might not represent the actual prevalence. Age is another factor; when age increases, the prevalence of ADHD decreases; the prevalence range in school-age children from 2.4 to 16.1%, and adolescents, it ranges from 2.2 to 9.9% (25).

The subtype of ADHD is also different among studies concerning the methodological differences. For example, the percentage of ADHD subtypes found in this study was in-attentive (10.8 %), hyperactivity-impulsivity (14.15%), and combined (25%). This displayed a difference in distribution compared to the studies that used the same tool, i.e., the studies from Ethiopia, Nairobi, and Trebizond reported the most common subtype was inattentive, hyperactivity-impulsivity, hyperactivity-impulsivity, and hyperactivity-impulsivity, respectively (9, 11, 21, 23). However, the combined subtype was the most common in our study, similar to Turkey (26).

This study also revealed that ADHD was also predicted by children's history of previous mental problems. Children with a history of last mental problems are almost nine times more likely to develop ADHD than children with no history of mental problems. This study is in line with the fact described in the synopsis of psychiatry 11^th^ edition books. Synopsis of psychiatry recognizes that comorbidity is a rule (27).

Moreover, children delivered by cesarean section had six times higher than to develop ADHD than their counterparts. Similarly, a study done in AL-Qalyubia primary school showed as caesarian section delivery was associated with ADHD. This might be due to caesarian section delivery will be predisposed the child to immune disorders. This may lead to altered immune maturation, increased exposure to the maternal microbial, and trauma in the brain (24, 28).

In this study, the odds of ADHD in children with a maternal history of substance use during pregnancy had nearly three times higher than sober mothers. This may be because substance use during pregnancy is not safe for the mother and the fetus (29, 30). As shown, the prevalence of ADHD in children 6 to 17 years old was high (13%). In addition, financial crises, children's previous history of mental problems, C/S delivery, and substance use were significant factors of ADHD. Therefore, it is better to implement a community-based ADHD screening and intervention program. In addition, recall bias might be encountered while interviewing the child's parent.
